# Appointment

**Published:** 1853-07

**Authors:** 


*Appointment.*—The vacancy in the chair of Theory and Practice of Medicine, in the Buffalo Medical College, caused by the resignation of the senior editor of this Journal, has been filled by the appointment of Dr. Thomas F. Rochester, of New York. It is believed that this arrangement will be one of favorable omen, both to Dr. Rochester and the school. We rejoice that this important chair is to be filled by one who contemplates a removal to this city, and will consequently feel that deeper interest in the honor and success of the school, which can hardly be felt to so great a degree by persons residing abroad, whatever their connection with it.

Dr. Rochester has already an enviable reputation as a lecturer. Originating in Western New York, he has enjoyed all the advantages of an excellent home education, and after a few years practice, of a long residence in Europe, and since that, he has occupied, in New York, the post of Physician to one of the principal dispensaries. He is at present lecturing, with acceptance, at the University School in that city, on a subject allied to the branch he is to teach in Buffalo.

We should also mention that Prof. Moore is now placed permanently on the staff of the school, as Prof. of Anatomy. Dr. Moore, long known as a successful teacher of surgery, seems destined to secure an equal reputation as an anatomist, and with his increasing familiarity with his subject, we predict an increased popularity to his already popular lectures. S. B. H.

